# Competing public narratives in nutrition policy: insights into the ideational barriers of public support for regulatory nutrition measures

**DOI:** 10.1186/s12961-022-00891-6

**Published:** 2022-08-09

**Authors:** Katherine Cullerton, Dori Patay, Michael Waller, Eloise Adsett, Amanda Lee

**Affiliations:** School of Public Health, Faculty of Medicine, The University of Queensland, 266 Herston Rd, Herston, QLD 4006 Australia

**Keywords:** Nutrition policy, Public attitude, Street intercept, Narratives, Framing, Narrative policy framework, Neoliberalism, Commercial determinants of health

## Abstract

**Background:**

Enacting evidence-based public health policy can be challenging. One factor contributing to this challenge is a lack of public support for specific policies, which may stem from limited interest or conviction by policy arguments. This can happen when messaging strategies regarding policy do not resonate with the target group and/or policy narratives compete in public discourse. To understand how policy messaging can better resonate with a target audience, we examined the frames and narratives used by the Australian public when discussing nutrition policies.

**Methods:**

We conducted 76 street intercept interviews in urban and regional settings in Queensland, Australia. Quantitative data were analysed using mean agreement scores and *t*-tests, and the qualitative data were analysed using an adapted qualitative narrative policy framework (QNPF). The QNPF is used to illustrate how competing narratives vary in the way they define different elements. These elements often include setting, characters, plot, policy solution and belief systems.

**Results:**

Level of support for all nutrition policies was generally moderate to high, although nutrition policies perceived to be most intrusive to personal freedoms were the least popular among the public. The value of fairness was consistently invoked when participants discussed their support for or opposition to policy. Using the QNPF, two distinct settings were evident in the narratives: concern for the community or concern for self. Villains were identified as either “other individuals, in particular parents” or “Big Food”. Victims were identified as “children” or “the food industry, in particular farmers”. Frequently used plots focused on individuals making poor choices because they were uneducated, versus Big Food being powerful and controlling people and the government.

**Conclusions:**

The study examined the frames and narratives used by the Australian public when discussing nutrition policies. By examining these frames and narratives, we gained insight into multiple strategies which may increase public support for certain nutrition policies in Australia.

**Supplementary Information:**

The online version contains supplementary material available at 10.1186/s12961-022-00891-6.

## Background

In the field of public health, scientists often struggle to translate evidence into policy [1]. One often-cited reason for this is lack of public support for a specific policy [2]. Particularly for interventions requiring regulatory or legislative change, perceived lack of public support is associated with politicians providing less support for such measures [3, 4]. This is of concern because regulatory policies are widely recognized as the most effective and equitable strategies in preventing noncommunicable diseases [5]. Therefore, it is vital to understand how public support for regulatory policies may be increased.

Low levels of public support for regulatory policy change can occur when the public is uninterested in, unconvinced by or opposed to policy arguments. This can happen when public messaging strategies do not resonate with the target group or when policy narratives compete in public discourse [6]. Such competition of policy narratives commonly occurs in the field of public health nutrition, particularly around regulatory policy actions, with food industry narratives frequently competing against public health narratives [7]. Large multinational companies producing and selling ultra-processed foods and drinks have been using strategic framing as a key strategy to build public and political opposition for regulatory nutrition policies for many years [8].

This contestation between ultra-processed food companies and nutrition advocates around policy action may be one reason that Australia has struggled to implement a range of effective regulatory or legislative measures in nutrition policy [9, 10]. Public health experts are now recognizing the need for successful counter-framing [11].

While competition between policy narratives commonly occurs in public health nutrition [3], we have a limited understanding of the public’s interest in or opinion of regulatory policy arguments, and importantly, whether current policy messaging strategies align with the target groups’ values and beliefs. Understanding this is important, as the way evidence and policy issues are discussed can make a difference in how people interpret and form an opinion about them. This concept is known as framing. Framing raises the salience of certain parts of a message via the presence or absence of certain words, phrases and images to provide thematically reinforcing clusters of facts or judgements [12]. Consequently, different frames can act as lenses through which to interpret information and have been promoted as one way to influence public opinion on policy issues [13]. However, people are not susceptible to just any frame. Audiences are more likely to be persuaded by frames from trusted sources [14] and those that correspond to their social reality and political ideology [15]. Most framing research has found that frames do not create new beliefs in people but rather trigger existing beliefs, values and memories in ways that reinforce the frame’s message [16–18].

How an issue is framed can dramatically affect the listener’s perception of the problem and whether it should be considered a public policy issue at all [19, 20]. However, for frames to be successful, they need to be communicated via tools that connect the different elements included in a frame in a meaningful way. Various methods are available to do this, such as using narratives [20–22]. Narratives help people understand and communicate information by organizing information in a way that is conducive to human cognition.

The development of effective narratives requires an understanding of the audience’s values, beliefs and attitudes. Numerous studies have found that people are more receptive to narratives that are congruent with their own world view [23–25]. The more a narrative appears to take place in a world populated with recognizable characters and language that look and function similarly to the audience’s world, the more engaging that story will be.

To understand how policy messaging can better engage a target audience, we examined the role of frames and narratives in the public acceptance of nutrition policies through street intercept interviews with urban and regional Australians. While public opinion surveys on public health nutrition issues have been conducted in Australia previously, most of these have been quantitative in nature [26]. Few studies in Australia have assessed levels of policy support alongside an examination of how individuals construct views on different nutrition policy options, and none have measured the level of policy support against the political persuasion/ideology of participants [26]. This is an important area to explore, as political ideology affects positions on specific policy issues [27]. While many voters do not have a clearly thought-out political ideology, they usually have ideological tendencies [28]. These general ideological tendencies have been shown to influence voting behaviour, which is of key concern to politicians making policy decisions [29].

Furthermore, public opinion studies frequently focus on urban participants, with many failing to engage participants from regional locations, a particularly important group when it comes to influencing policy [26]. The aim of this study was thus twofold: firstly, to understand how the level of support for each policy varied by location and voting behaviour; and secondly, to gain unique insights into the values, beliefs and perceptions of the general public concerning public health nutrition policies. This knowledge will assist health communicators/advocates in better framing complex issues to increase audience receptivity and expand our understanding of the ways public support can be forged for evidence-based regulatory nutrition policies.

## Methods

This study used a mixed methodology, applying an interview tool involving survey questions and open-ended questions within a street intercept interview method.

### Theoretical approach

Framing theory provided the overarching theoretical framework for this study. We specifically used elements of the qualitative narrative policy framework (QNPF) [30] to inform the data analysis. The QNPF is an extension of the Narrative Policy Framework [6], which was developed in 2005 and recognizes that narrative plays a central role in human cognition and communication—that is, people prefer to think and speak in story form. The QNPF offers a systematic approach to understanding the role of narratives in the policy process and illustrates how competing narratives vary in the way they define different structural elements [30]. These elements often include setting, characters, plot, policy solution and belief systems (Table 1) [30]. QNPF, as an analytical tool, enables systematic analysis of the ways strategic framing has been used and internalized in the discourse around regulatory nutrition policies.

**Table 1:** Narrative elements based on QNPF

Element	Definition
Setting	The setting is the space where the action of the story takes place over time (often contextual)
Characters	Actors are often seen or described as “victims that are harmed by the problem, villains that intentionally or unintentionally cause the harm and heroes that provide or promise relief from the harm” [31]
Moral of the story	The policy solution promoted by a policy narrative
Plot	Plots explain the connections between the elements of the narrative [31]
Belief systems	Ideologies and beliefs based on what individuals perceive as their reality

The QNPF has been successfully applied previously in public policy studies examining strategic framing and public attitudes towards proposed regulatory changes; however, this is the first time the QNPF has been applied to nutrition policy [30, 32].

### Data collection

We were interested in capturing the views of those who do not usually participate in relevant surveys and may have been under-represented in previous public opinion surveys. Accordingly, we decided to undertake street intercept interviews, which involve approaching potential participants as they go about their daily lives in a neighbourhood and inviting them to participate in an interview. This method has numerous benefits, including its suitability for accessing a broader range of individuals across demographic categories than traditional sampling permits, and higher response rates and less bias than encountered with online, mail and telephone surveys [33, 34].

The setting for the interviews involved the main shopping streets of an urban location (Brisbane City, Queensland, Australia population: 2,271,000) and the main shopping street and the adjoining parks of four regional locations in Queensland (Miles, Roma, Chinchilla, Toowoomba, with populations ranging in size from 1800 to 114,000) [35]. These locations were chosen to represent a major city, inner regional, outer regional and remote locations [36], and ethics approval was obtained (#2019001612).

From September to November 2018, three trained interviewers recruited participants in the five locations. Any people who were alone and were standing still, sitting or appeared to have the capacity to stop (e.g. those strolling by) were approached and asked if they would like to participate in an interview. In the city location, sometimes people would approach the interviewers and ask about the study. When this occurred, the interviewer explained the study and invited the individual to participate. English-speaking adults (≥ 18 years) who were Australian citizens were eligible to participate. Recruitment continued until data saturation was reached (i.e. the interviews offered no further new insights).

All interviews took place during daytime hours, though the time of day varied. Their duration ranged from 4 to 22 minutes, with the average being 8:30 minutes. They were conducted at the time and location where the participant was initially approached and were audio-recorded with the participants’ consent.

Altogether, eight nutrition policy measures were discussed in the interviews: banning vending machines selling unhealthy food or drinks in schools; implementing a tax on sugar-sweetened beverages; banning advertising of junk food targeting children during popular TV viewing times; subsidizing the sale of fruit and vegetables; media campaigns to encourage people to eat healthier foods; encouraging food companies to provide food labels that carry clearer information about the nutrition content of foods; product reformulation to contain less salt, sugar and saturated fat; and freight subsidies from the government for transport of healthy food to remote Aboriginal communities. These policies were selected as they have been the most commonly investigated measures assessed in earlier public opinion studies in Australia [26].

The semi-structured interview tool included quantitative and qualitative components, including questions about participants’ level of agreement with different nutrition policies, and demographic and voting behaviour questions. The qualitative component of the interview asked participants *why* they agreed or disagreed with each policy. Probing questions were asked to expand upon the thoughts and experiences of participants. The wording of the initial questions was specifically designed to provoke an emotional response and initiate critical reflection in participants by using strong, emotive language, such as “*make* companies do something” and “*ban* advertisements”. The detailed list of questions is provided in Additional file 1.

### Data analysis

#### Statistical analysis

Mean agreement scores were calculated for each policy question. These ranged from a minimum of 1 to a maximum of 5 (highest agreement). Additionally, *t*-tests were used to compare agreement scores between location (urban, regional) and political groups (conservative, undecided, progressive) based on the a priori hypotheses where we expected there to be differences between those with different political views, and between those from urban and regional locations. Political views were assigned as follows: “Conservative”, votes for Liberal Party, National Party or One Nation Party; “Progressive”, votes for Labor Party or Greens Party; “Undecided”, does not vote for a particular party, unsure whom they vote for. As only one participant voted Independent, it was decided to include them in the “Undecided” category. Geographical classification was determined using the Accessibility Remoteness Index of Australia Plus and participant’s postcodes [37]. Socioeconomic status of participants was determined using the Index of Relative Socio-economic Disadvantage of the Socio-Economic Indexes for Areas score for their postcode [36]. Statistical analyses were undertaken in Stata 15.1 (StataCorp LLC).

#### Qualitative analysis

Interviews were de-identified, transcribed and uploaded to NVivo version 11. A coding framework based on the Narrative Policy Framework was developed which incorporated the elements of “the setting”, “the hero”, “the villain”, “the victim”, “the moral of the story” and “the plot”. KC and DP separately read each interview line by line and coded according to the coding framework, and inductively coded for additional issues, including the values and beliefs of the participants [38]. Any disagreements were discussed. Codes developed inductively outside the initial coding framework were further analysed into sub-themes and then refined into themes [38].

## Results

### The sample

**Fig. 1:**
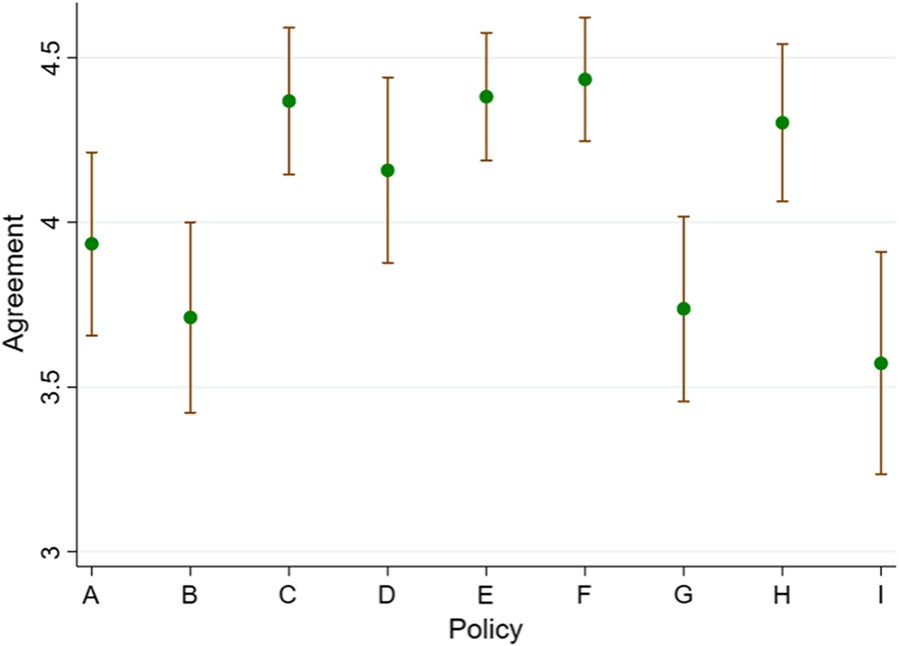
Mean agreement by policy with 95% confidence interval. **a** Ban vending machines selling unhealthy food or drinks in schools. **b** Impose a tax on manufacturers for the high-sugar drinks they sell. **c** Ban advertising of junk food targeting children during popular TV viewing times (including 6–9 pm). **d** Subsidize the sale of fruits and vegetables, making them cheaper for consumers. **e** Conduct media campaigns to encourage people to eat healthier foods, like fruit and vegetables. **f** Encourage food companies to provide food labels that carry clearer information about the nutrition content of foods. **g** Make companies reformulate foods to contain less salt, sugar and saturated fat. **h** Provide freight subsidies from the government for transport of healthy food to remote Aboriginal communities. **i** Introduce a 20% tax on sugary drinks that would increase the price for consumers

In total, 76 people participated in the street intercept interviews; demographic characteristics are presented in Table 2. The participants’ level of support for the nutrition policies is provided in Table 3 and summarized in Fig. 1.

**Table 2:** Demographic characteristics (*n* = 76)

	No.	%
Sex		
Male	29	38.2
Female	47	61.8
Age range (years)		
18–24	17	22.4
25–44	19	25.0
45–64	23	30.3
65+	17	22.4
Education		
Below year 12	17	22.4
Year 12 or diploma	22	28.9
Bachelor’s degree	25	32.9
Master’s degree	12	15.8
Location		
Regional	34	44.7
Urban	42	55.3
Socioeconomic status		
1–4 low	22	28.9
5–6 medium	24	31.6
7–10 high	30	39.5
Political party		
Progressive	22	28.9
Undecided	20	26.3
Conservative	34	44.7
Main food shopper		
No	18	23.7
Yes	38	50.0
Shared	20	26.3
Live in a household with a child under 18		
No	51	67.1
Yes	25	32.9

**Table 3:** Mean agreement scores for each policy by location and political views (results based on *t*-tests between groups)

	Policy A	Policy B	Policy C	Policy D	Policy E	Policy F	Policy G	Policy H	Policy I
	Ban unhealthy vending in schools	Tax high-sugar drinks	Ban junk food ads (kids viewing)	Subsidize fruits and veg.	Media campaigns fruit and veg.	Clearer food labels	Reformulate foods	Freight subsidies Aboriginal communities	20% tax on sugary drinks
	Mean (SD)								
Total	3.9 (1.2)	3.7 (1.3)	4.4 (1.0)	4.2 (1.2)	4.4 (0.8)	4.4 (0.8)	3.7 (1.2)	4.3 (1.0)	3.6 (1.5)
Location									
Rural	4.2 (1.2)	3.6 (1.3)	4.4 (1.1)	4.2 (1.2)	4.3 (0.9)	4.4 (0.8)	3.9 (1.1)	4.0 (1.3)	3.4 (1.5)
Urban	3.7 (1.2)	3.8 (1.3)	4.4 (0.9)	4.1 (1.3)	4.5 (0.8)	4.5 (0.8)	3.6 (1.3)	4.5 (0.8)	3.8 (1.5)
Difference (rural/rural − urban)	0.4 (−0.1, 1.0)	−0.2 (−0.8, 0.4)	−0.02 (−0.5, 0.4)	0.1 (−0.5, 0.6)	−0.2 (−0.5, 0.2)	−0.1 (−0.5, 0.2)	0.3 (−0.2, 0.9)	−0.5 (−1.0, −0.1)	−0.4 (−1.1, 0.3)
*P* value	0.12	0.57	0.90	0.76	0.42	0.44	0.27	0.02	0.24
Political views									
Progressive	4.2 (1.1)	4.0 (1.3)	4.6 (0.8)	4.4 (1.0)	4.8 (0.4)	4.5 (0.7)	3.8 (1.2)	4.5 (0.8)	4.0 (1.3)
Unsure/Independent	3.8 (1.2)	3.5 (1.5)	4.4 (0.9)	4.5 (1.0)	4.4 (0.8)	4.7 (0.8)	3.9 (1.2)	4.4 (0.9)	3.7 (1.6)
Conservative	3.8 (1.3)	3.6 (1.1)	4.2 (1.1)	3.9 (1.4)	4.1 (1.0)	4.3 (0.9)	3.6 (1.3)	4.1 (1.2)	3.2 (1.5)
U/I vs Prog. 95% CI	−0.4 (−1.2, 0.3)	−0.6 (−1.5, 0.3)	−0.2 (−0.8, 0.3)	0.1 (−0.6, 0.7)	−0.5 (−0.9, −0.1)	0.2 (−0.3, 0.6)	0.03 (−0.7, 0.8)	−0.1 (−0.7, 0.4)	−0.3 (−1.2, 0.6)
*P* value	0.25	0.17	0.37	0.79	0.02	0.53	0.93	0.59	0.47
Con. vs Prog. 95% CI	−0.4 (−0.3, 1.1)	−0.4 (−1.0, 0.2)	−0.4 (−0.9, 0.2)	−0.5 (−1.2, 0.2)	−0.7 (−1.1, −0.3)	−0.2 (−0.7, 0.2)	−0.2 (−0.9, 0.5)	−0.5 (−1.0, 0.1)	−0.8 (−1.5, 0.01)
*P* value	0.23	0.21	0.20	0.15	0.002	0.30	0.56	0.13	0.05
Con. vs U/I 95% CI	0.02 (−0.7, 0.7)	0.2 (−0.5, 0.9)	−0.1 (−0.7, 0.5)	−0.6 (−1.3, 0.1)	−0.2 (−0.8, 0.3)	−0.4 (−0.9, −0.1)	−0.2 (−0.9, 0.5)	−0.3 (−0.9, 0.3)	−0.4 (−1.3, 0.4)
*P* value	0.95	0.58	0.70	0.1	0.37	0.11	0.52	0.33	0.31

We could not determine a response rate for the city location, as several individuals approached our interviewers and asked to participate. However, for the regional locations, the response rate was 89%, with only four individuals (three male, one female) refusing to participate. The demographic characteristics were relatively evenly distributed, although females and conservative voters were overrepresented in the sample (Table 2).

### Quantitative results

The level of support for all nutrition policies was moderate to high (Table 3 and Fig. 1). All policies had a mean level of support greater than 3.5 (scale ranged from 1 to 5, with 3 as the scale midpoint). The highest levels of support were for banning junk food ads during children’s viewing times (4.4), conducting a media campaign promoting fruit and vegetables (4.4), providing clearer food labels (4.4) and providing freight subsidies for healthy food for remote Aboriginal communities (4.3). The lowest levels of support were for taxing companies that make high-sugar drinks (3.7), making companies reformulate foods to reduce sugar and salt (3.7), and a 20% consumer facing tax on sugary drinks (3.6).

There were no consistent differences in the mean level of support for policies between urban and rural participants, although rural participants were less likely to support freight subsidies for Aboriginal communities than urban participants (−0.5 95% CI [−1.0, −0.1], *p* = 0.02). Support for all policies was generally highest among progressive voters and lowest among conservative voters, but only two of these differences approached statistical significance. Conservatives were less in favour of media campaigns to promote healthier foods (−0.7 [−1.1, −0.3], *p* = 0.002), and less in favour of a 20% tax on sugary drinks than progressive voters (−0.8 [−1.5, 0.01], *p* = 0.05). The undecided group generally had an agreement score between the progressive and conservative voters. However, this undecided group did report the highest agreement for subsidizing fruit and vegetables, clearer food labels and reformulating foods (although differences were not statistically significant).

### Qualitative results

**Fig. 2:**
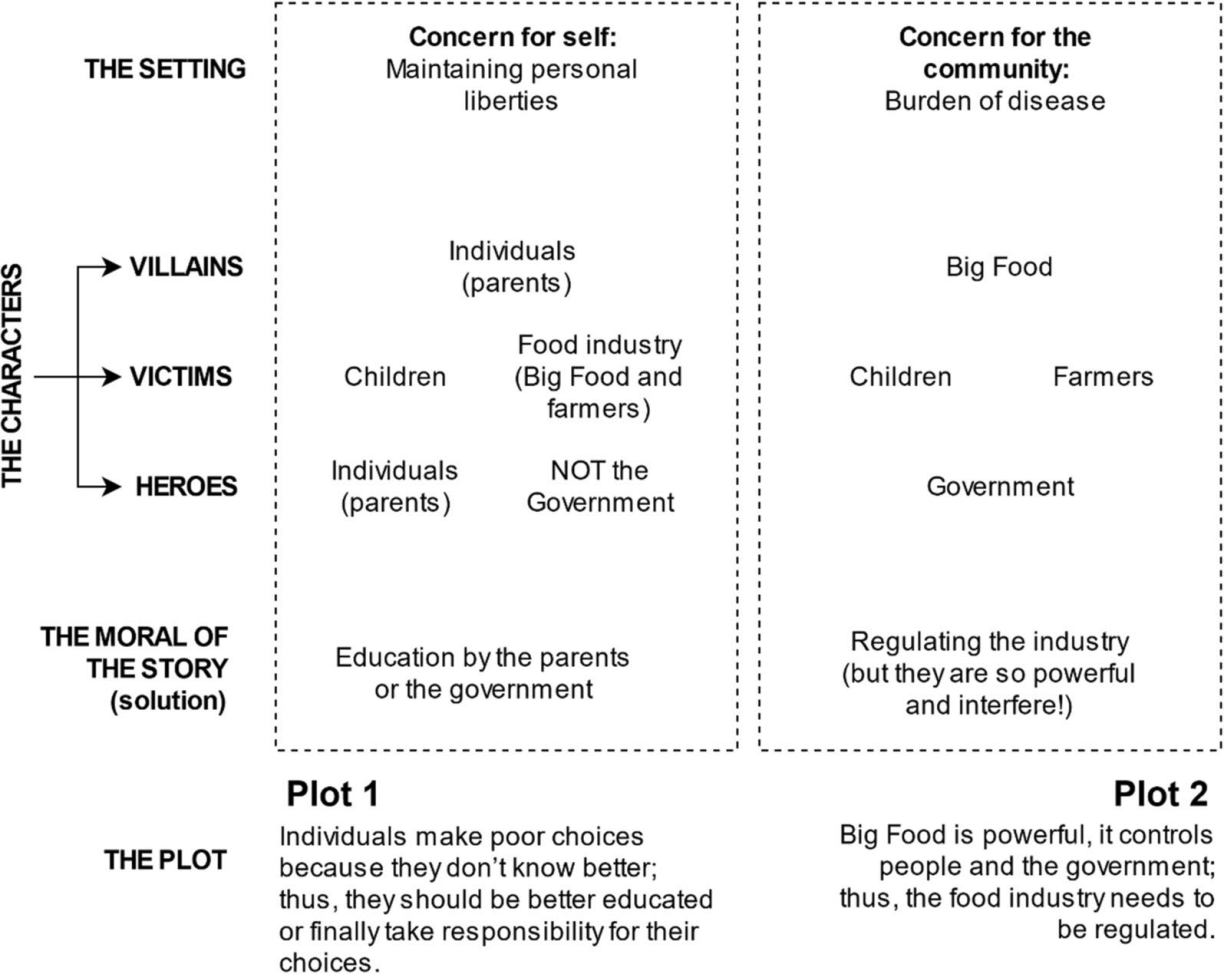
Elements of the narratives in interviewee data

The qualitative data provided insight into why people agreed or disagreed with a particular nutrition policy. By drawing on the QNPF coding framework, we were also able to identify the setting, key characters, the plot and the moral of the story in the overall responses (Fig. 2). Most people interviewed either had limited knowledge of the policies we mentioned or said they had never thought about the issues or were ambivalent towards them. Some also changed their position as they went through the interview process.

#### The setting

For most participants, their explanation for why something should or should not happen was couched in the context of two distinct settings: *concern for the community* or *concern for self*. For some, there was overlap between these two settings depending on the policy issue discussed.

##### Concern for community

Several elements were mentioned under concern for the community. One concern frequently raised was around the “obesity epidemic” and its impact on society and the health system:

*There’s an epidemic around people getting too overweight, and it’s a burden on the health system, so I think to reduce this burden, governments need to get involved in informing people about how to have better understanding about how to eat more healthily.* [Male, 45–64 years, urban, progressive]

##### Concern for self

A significant number of participants noted the importance of freedom of choice and the agency of individuals when reflecting on the different nutrition policy options.

*I’m maybe convinced that there are certain things that we could look at as long as it doesn’t take away personal freedoms. Which to me would become rather unjust, and we'd be going towards police state sort of stuff.* [Male, > 65 years, urban, progressive]

In both contextual settings, the importance of fairness towards consumers and the industry was often cited by the participants. However, fairness was seen in different ways by different participants. Some invoked fairness in terms of equality of distribution, others in terms of equality of opportunity, and finally, some spoke of needs-based fairness.

#### Characters

##### Villains

Participants easily identified villains when discussing the nutritional health of the country. Most commonly, participants either blamed “others” (individuals other than themselves) for making unhealthy dietary choices or blamed the food industry for influencing the public and the government.

###### “Others”: Individuals, parents, teachers

The most common villains identified by participants were “others” because of their “lack of self-control” and “lack of knowledge”.

*It’s apparent that some people aren't very good at making those decisions themselves.* [Female, 25–44 years, urban, undecided voter]

*There's a lot of people who just don't know how to do things or how to eat properly.* [Male, 25–44 years, urban, progressive]

Interestingly, parents (often mothers) and teachers were frequently blamed, with recurrent references to children missing out on learning about healthy food and cooking skills and parents with permissive attitudes towards their children’s food choices.

*I think it comes back to schools. Gone are the days of home economics, of teaching them to cook… I know that it's a horrible generalization, but I do believe that teachers don't care as much as they used to.* [Female, 45–64 years, regional, conservative]

*Obviously something is really missing from this lot of parenting, our generation, we've got the hugest obesity problem we've ever had. […] It’s not just the foods that they're eating, it's the quantity that they're allowed to eat.* [Female, 45–64 years, regional, undecided voter]

###### Big Food

Several people identified the food industry as a villain; in this context, they mostly referred to multinational companies responsible for producing ultra-processed foods (Big Food) rather than local producers.

*McDonald's and large corporations who are kind of setting the scene of what food is appropriate to eat on a mass scale.* [Male, 25–44 years, urban, progressive]

Primarily industry marketing and advertising activities of ultra-processed foods were highlighted as harmful activities, but some participants also flagged the power of large food corporations over the government, the public and the food supply chain.

*I'd close Woolies and Coles. […] There's supposed to be an investigation into the duopoly of control that they have, because they are butchering what's happening through farming.* [Male, > 65 years, regional, conservative]

##### Victims

Participants most commonly identified children and the food industry, especially farmers, as the victims in their narratives.

###### Children

Children were often described as victims of inappropriate parenting or teaching practices, as mentioned previously, or of food industry advertisements targeting them. Their vulnerability to these practices was a concern for many.

*I think children are such an easy target for advertisers for so many products, and I don't think advertising should be aimed at children at all, for anything.* [Female, 25–44 years, urban, undecided voter]

*When we're talking about children who are minors and passive receivers of messages, it's harder to say “they have a choice to not view it, or to leave the room” or whatever. I think that they’re quite a captive audience.* [Female, 45–64 years, urban, progressive]

###### Food industry

Some participants perceived the food industry as benign actors, doing their job but also benefiting society. When discussing the food industry, participants referred to a wide gamut of actors ranging from sugar-sweetened beverage manufacturers and fast-food companies to farmers. The principle of fairness was frequently invoked when discussing this group, as well as a sense that they should be protected and not disadvantaged for just “doing their job”. Interestingly, this sentiment was expressed by a range of participants regardless of their political party preference or geographical location.

*I think that's just picking on one industry, which would not be readily agreed to by a lot of people. It would ... not only would it be unfair to that industry, but it would cause a ruction between that industry and another industry.* [Male, > 65 years, urban, progressive]

*I don't think that ... I think it's their product, so I don't think that they should be taxed based on what they produce for the high sugar content. Because at the end of the day, they've gotta make profit somehow, and if everyone's enjoying it, I guess they can keep it.* [Female, 18–24 years, urban, undecided voter]

Other participants highlighted the important benefits of industry sponsorship to society. This ranged from sponsorship of sporting events and schools to local community events.

*Whilst junk food is bad, and you get pester power unfortunately at the moment, it's the junk food companies that do have the money, and where they provide funding in other places are incredibly beneficial. […] If all of that funding got pulled, then a lot of that sporting would actually disappear completely. That's where I think it's a balance, especially from home, of treating your kids that this is special occasion food versus banning it completely.* [Female, 45–64 years, urban, progressive]

###### Farmers

Participants across our sample periodically expressed their concern that farmers as food industry actors may be negatively affected by regulatory nutrition policies, and that if this was the case, they would not support the policy.

*Providing that we get a decent outcome for the farmers and for the people that are producing it? Yes.* [Female, 45–64 years, regional, undecided voter]

*I wouldn't want farmers missing out, so providing the primary producers are looked after and it's the government who's absorbing that cost difference, that's what I would care about*. [Female, 45–64 years, urban, progressive]

##### Heroes

The primary heroes identified by participants were individuals who take responsibility for their own choices, parents and the government.

###### Be your own hero

As mentioned earlier, many participants expressed the belief that individuals should start taking responsibility for their dietary choices. For these participants, taking personal responsibility for food choices was of paramount importance:

*There has to be a time when people look after themselves and they take responsibility for their own actions, which includes eating.* [Female, > 65 years, regional, conservative]

*I honestly think that everyone should be accountable for their own food choices.* [Female, 24–44 years, regional, conservative]

###### Be a better parent

Participants frequently perceived parental education of their children, particularly for mothers, to be the foundation of behavioural and lifestyle choices. Therefore, parents were seen as the hero of the narrative if they guided their children towards healthy habits.

*I think it's about time that we went back to mum showing the child and growing food with the child and showing them what are the correct things to eat and food to cook, instead of takeaways.* [Female, > 65 years, urban, conservative]

###### Government, our hero

Most participants clearly saw a role for the government in solving society’s nutrition-related problems. Participants expressed the belief that the government had a responsibility for the health of the population, and that it should be in charge of leading a response. These participants mostly identified as progressive party supporters; however, some undecided and conservative voters also saw a role for the government to lead, particularly for education-based programmes for the population.

*I think the government is elected by the people, and it is their responsibility, it's their charter and it's their right to create policies that give people the best chance to live long and healthy lives.* [Female, 45–64 years, urban, progressive]

*With our rising obesity rates, I think that it needs to come from government, higher up, right down through to change.* [Female, 25–44 years, regional, undecided voter]

###### Back off, Big Brother

However, there was also the sentiment, particularly among regional conservative participants, that they did not want the government to step up as a “hero” because of a lack of trust in its ability for effective administration. There was also confusion from some participants as to what the government could feasibly do to intervene in this arena.

*Don't let government organize it. Couldn’t organize a chook raffle.* [Male, > 65 years, conservative]

*In terms of government interference in private markets, they can't really do it, they can't restrict every private company*. [Female, 18–24, urban, progressive]

Others were vehemently opposed to government involvement and invoked the so-called nanny state or Big Brother argument, stating that the government should not interfere with personal liberties over lifestyle choices.

*You hear things on the news, the government now is going to ban some schools from doing something, and the first thing you say is, “Why is the government is doing that? Why aren't the school and the parents doing that? Why suddenly aren't people allowed to make up their own rules and regulations?” […] Children have parents. They make those decisions—well, they should.* [Female, > 65 years, regional, conservative]

*The government, I don't have much faith in them of late. But I think that's up to the individual, really. It's the government's responsibility to have a lot of choices available. At the end of the day, you've got to be responsible for whatever choice you make, whether it's food or whatever.* [Female, 45–64 years, regional, conservative]

#### Moral of the story

##### Providing information to support individual decision-making

Health education was most commonly cited as the best solution for improving the nutritional status of the population. Participants were comfortable with a range of actors taking on this responsibility.

*Conduct media campaigns to encourage people to eat healthier foods like fruit. Oh, I strongly agree. Because that's education about what’s good for you, and that's informing people so they can make their own decisions rather than imposing on them.* [Female, > 65 years, urban, conservative]

Some people saw this need particularly in school, which was in line with where most victims are located and where people should be educated according to the narrative:

*The fix has got to be coming back to schools and teaching these dopey people of today.* [Female, 45–64 years, regional, conservative]

Again referring to the importance of personal responsibility and building personal skills and knowledge, some participants highlighted that instead of regulatory nutrition policies, people should be better informed to make healthier choices.

*Make companies reformulate foods? No, because that's Big Brother. If they label, then people can choose for themselves.* [Female, > 65 years, urban, conservative]

Following the same narrative, there were high levels of support for improving food labelling on food packets, with the common sentiment being “if people knew what was in food, they would make healthier choices”.

*Just have easier-to-consume information so you don't have to understand, what is sodium, what is sugar, what is the different kinds of things. So simplifying that I think would make sense.* [Male, 18–24 years, urban, undecided voter]

##### Regulating the food industry

While education of the public was the most common response amongst the participants when considering how to improve the nutritional status of the population, some acknowledged that instead of public education, food industry regulation was necessary to improve health status. This sentiment was mostly predominant amongst progressive voters; however, some conservative and undecided voters also agreed:

*To police how people feel about health and how they implement it; I think that just sounds unrealistic. I think what's probably more effective if they regulated the people that are selling food.* [Female, 45–64 years, regional, undecided voter]

*Make companies reformulate the foods… I'm gonna go strongly agree for that. I think that's a good idea. Making companies more responsible for what they're putting out.* [Male, 25–44, urban, conservative]

However, many interviewees, particularly progressive voters, were sceptical that the food industry could be regulated, due to industry interference in policy-making.

*Make companies reformulate foods, I doubt that'll happen. Because those companies are too powerful.* [Female, 45–64 years, regional, progressive]

*It's the big corporations… the two or three companies, like Nestle, Cadbury's, Coke, which own all of the various subsidiaries, down food chain, down to our cheese and our bread and all those sorts of things, who are multinational companies that have control over the policy.* [female, 45–64 years, urban, progressive]

##### Making unhealthy foods and drinks more expensive or unavailable

Policy measures that had a more direct constraining impact on consumers, such as increasing the cost of unhealthy foods or banning their sale in vending machines, were generally seen as intrusive and had lower support from participants.

*I don't believe the sugar tax will go back to the manufacturer. And what does the government do with the sugar tax? It doesn't benefit the provider being off the farm, it doesn't help anyone on the farm at any point. So all drinks should be costed the same for the freedom of choice and people can make their own choices.* [Female, 45–64 years, regional, conservative]

*I think a total ban is too controlling and too Big Brother-ish*. [Female, 45–64 years, urban, progressive]

##### Making healthy food cheaper

Many participants cited the fact that healthy food is more expensive than unhealthy foods, and thus subsidies were seen as a popular, potential solution to this problem.

*The sales of fruits and vegetables, making them cheaper for consumers. Yes, that would entice people to buy more, absolutely.* [Male, 25–44 years, regional, conservative]

This understanding of the impact of cost on food purchasing behaviour extended to most participants supporting freight subsidies in remote Aboriginal communities, although several regional participants stated that the subsidies should be available to disadvantaged regional communities as well.

*There's a lot of dollars in transport miles and a lot of Aboriginal communities are in desert land. There's a lot of rural Aboriginal communities who are so remote that it's really hard to get the fruit and veggie growing locally, so let's get them there affordably.* [Female, 45–64 years, urban, progressive]

#### The plot

##### Plot 1: individual responsibility to make better personal choices

Most participants believed that people were consuming unhealthy foods and beverages because of their lack of knowledge about healthy choices or their lack of concern about the harmful impact of a poor diet.

*Obesity is on the rise. And obviously, if it's on the rise then we're struggling to manage ourselves really.* [Male, 25–44 years, urban, conservative]

*If people understand that eating salt, sugar and saturated fats can have a long-term effect on your body, if that's clearer to people as they're growing and having families, then they can educate their children. I think that might help.* [Male, 25–44 years, regional, conservative]

This plot closely resonates with the idea of personal liberties and individual responsibility, stemming from neoliberal ideologies, pointing the blame to individuals (usually to “others” rather than themselves). Accordingly, the solution to the villains’ harmful actions lies in the plot: the public needs to be educated, which, according to many participants, can be driven by the government, parents or teachers.

##### Plot 2: commercial determinants of diet

The other, albeit less common, plot focused on the food industry, in particular transnational corporations, as villains. This was due to participants recognizing its role in heavily advertising its unhealthy products and negatively influencing nutrition policy-making.

*I'm going back 60 years, to what I did as a kid and the way I was brought up. We just knew that you didn't eat cakes and lollies and all that stuff just for the want of doing it. Of course, there wasn't any takeaway food then, really. So, that's where the problem comes in, takeaway food, to a big degree. And they brainwash people to go and buy whatever, and the kids want it. So they need some laws to help people along.* [Female, > 65 years, regional, conservative]

Based on this plot, the solution would be tighter industry regulation, particularly around unhealthy food advertisements.

In summary, the qualitative data revealed two major narratives prevalent in the sampled Australian population. The first narrative finds individuals at fault for not making the right, healthy dietary choices, rooted in their perceived lack of knowledge about unhealthy and healthy foods. Consequently, they should start taking responsibility for their lifestyle choices, and this should be supported by education programmes provided by the government. According to the second narrative, the food industry has too much influence over people’s dietary habits; therefore, regulatory nutrition policies are needed. However, implementing such measures will be challenging due to the power of the food industry and its ability to interfere with policy-making.

## Discussion

### Results in relation to other findings in Australia and internationally

This study found that the level of support for all eight nutrition policies was moderate to high, with most participants supporting banning of junk food ads during children’s viewing times, conducting media campaigns promoting fruit and vegetables, providing clearer food labels and providing freight subsidies for remote Aboriginal communities. The lowest levels of support were for taxing companies that make high-sugar drinks and making companies reformulate foods to reduce sugar and salt. We found that the geographical location of participants had limited impact on their attitude towards nutrition policy. This aligns with another study suggesting that sociodemographic variables have a minor impact on public attitude towards health policies [39].

One factor which did make a small difference in levels of agreement was political affiliation, with support for all nutrition policies generally highest among progressive voters and lowest among conservative voters. The undecided group generally had an agreement score between those of the progressive and conservative voters. This corresponds with a prior study suggesting that political affiliation shapes public acceptance of public health measures [40], and several other studies reporting the same for public policies in general [41–43]. However, the qualitative results revealed that the political affiliation of participants did not always produce consistent results regarding support for the various nutrition policies. For example, several conservative-voting participants expressed their dislike of government intervention but then endorsed regulatory nutrition policies banning advertisements or vending machines promoting junk food for children. This lack of coherence is not unusual in policy support [29]. While political orientation can partly explain an individual’s agreement or disagreement for different policies, core values seem to play an equally important role [29]. Conflict in an individual’s core values is one explanation as to why an individual could take opposing views on two related policies [44]. For example, someone might wish for equality due to their inherent egalitarian values, but this may conflict with their strong ideas about self-reliance, ultimately resulting in limited support for government programmes aiming to improve equality.

### Values, beliefs and perceptions invoked compared with previous literature

The QNPF proved to be a useful analytical tool for understanding the narratives behind public attitudes towards regulatory nutrition policies. The quantitative analysis demonstrated that the public was most supportive of policies which rely on strategies to educate consumers through media campaigns, labelling products, and protecting children by banning junk food advertisements targeting them. The qualitative analysis explained this trend by highlighting that most people believe that nutritional knowledge and personal responsibility are the main drivers of dietary choices. These findings accord with those of prior studies [45–49] and may reflect the effectiveness of the food industry strategic framing to oppose regulatory nutrition policy measures.

When participants disagreed with regulatory measures, such as taxes on sugar-sweetened beverages and product reformulation, their narratives were mostly driven by scepticism towards government interference in personal liberties. Also of concern was the perceived effectiveness of these measures, and whether any gained revenue would be allocated for public purposes. Other studies have confirmed such considerations [45, 47–49]. Our quantitative data indicate that measures restricting personal liberties were the least popular among the interviewees, as seen in prior studies [26]. However, it was noted that several participants who were initially against increasing any form of government control changed their mind after further deliberation on policy measures, resulting in reasonably high acceptance of the assessed policies within our sample. This resonates with Mettler’s [44] proposition that individuals may be philosophical conservatives but utilitarian liberals. This suggests that when people are asked broad questions about government involvement, they reflexively respond in a more conservative manner, but when asked more specific questions about funding for specific initiatives, for example, unemployment insurance, they become more progressive [44].

Within public health, it is well known that health education initiatives are unlikely to change dietary behaviour if applied in isolation and not as a part of a comprehensive set of supply- and demand-side measures [5]. However, our analysis revealed that the role of individual responsibility dominated people’s thinking about dietary choices, which in turn informed participants’ attitudes towards the proposed policy solutions. Prior studies have found that lack of knowledge and personal responsibility are perceived to be the main reasons for poor nutrition within society, which should be remedied by public education programmes [46, 48–51]. Unsurprisingly, the personal liberties and individual responsibility frame is used heavily and promoted by the food industry [52], and our data demonstrated the diffusion of this narrative.

While blaming individuals and their lack of education about nutrition was a popular response, there was limited mention of the broader structural determinants of health other than education, income levels and geographic location. More specifically, there was limited recognition of the commercial determinants of health, with participants most commonly recognizing the marketing and advertising strategies that companies undertake; only a few talked about industry interference with policy-making. In general, participants did not mention the other ways the food industry shapes population diet and the benefits of regulating industry activities. Similar findings were reported in other studies [47, 50].

Our finding that children were often perceived as victims corresponds to earlier studies [47, 49]. Children may be viewed as requiring protection because they do not have the same agency as adults to practise individual responsibility; therefore, parents are often seen as responsible for their actions and decisions [53].

Interestingly, the food industry, including sugar-sweetened beverage manufacturers, were often perceived by participants to be the victims of regulatory nutrition policies, with the value of fairness frequently invoked. This suggests that corporate social responsibility and community sponsorship schemes may have succeeded in positively promoting various companies within the food and beverage sector. The withdrawal of the state (deregulation) and celebrating free markets and personal liberties are cornerstones of neoliberal thinking [54]. Regulatory nutrition policies go against these ideologies, resulting in the food industry being seen as the victim of government regulation and being punished unfairly while doing their job. The tendency of some participants to sympathize with the food industry and not acknowledging any harmful corporate activities, while at the same time being deeply suspicious about any regulatory measures by the government, demonstrates the tension between these two concepts.

Importantly, much of the concern regarding food industry actors was for the livelihood of farmers. This trend is not explained by neoliberal ideologies, but resonates with the public on a different level. Studies have shown that Australians consider rural industries and regions to be very important to the nation’s future [55, 56]. Furthermore, many Australians believe that farmers are imbued with qualities such as resilience, strength and thrift, and a “favourable social and moral status” not enjoyed by their city-dwelling counterparts [55]. This may be due, in part, to the nature of media images and discourses seen and heard by the public that rely upon romanticized notions of food and farming [57]. Alternatively, there is increasing acknowledgement of the importance of farming to the notion of sovereign food security, and this may also contribute to these favourable attitudes.

### Policy implications

The QNPF proved to be a useful analytical tool for this study because it helped to understand the ways that strategic framing shapes public attitudes towards nutrition policies, by explaining and connecting elements of participants’ narratives. We identified several key findings to inform the development of more robust frames for nutrition policies which may help counterbalance the strategic narratives of the food industry to oppose evidence-based regulatory nutrition measures. We confirmed that tax policies were the least favoured measure by the public. This is important for future research because tax policies can be used as a benchmark for testing the success or failures of future frames.

Few participants acknowledged the role of the food industry in shaping food choices for society. It was not uncommon for participants from both ends of the political spectrum to feel sympathy for the food industry encountering possible regulatory policy. This suggests three courses of action may be required.

Firstly, those involved in communicating information about nutrition policy need to shift the language used away from the dominant framing of individual responsibility. While people continue to perceive themselves to be solely responsible for their dietary choices and disregard the broader determinants of health (such as commercial factors), governments will continue to respond to this by implementing policies predominantly focused on education [58]. The second course of action is for advocates to consider, in the short term, the use of more benevolent language towards business when discussing regulatory nutrition policies. This may be aided by incorporating the value of “fairness” into narratives. Finally, framing policies as providing benefits to groups perceived as victims could be a worthwhile strategy for garnering public support; in particular, highlighting children or farmers as the beneficiaries of planned nutrition measures could improve public acceptance.

While undertaking these changes in policy narrative may result in a public more receptive to evidence-based nutrition policy, it is important to note that there will always be the opportunity for a counter-narrative that portrays advocates themselves as “lying villains”.

## Strengths and limitations

A key strength of this study was the focus on understanding the dominant values, beliefs and narratives used by the public regarding nutrition policies, an under-researched area in public health [26]. However, utilizing the QNPF to categorize underlying assumptions and beliefs of participants may not have truly captured broad-ranging beliefs that a fully inductive analysis—for example, using grounded theory—may instead have captured. Further, our conclusions around voting behaviour and policy support may have been strengthened by quantitatively collecting and incorporating values and world view data.

Importantly, the street intercept interview method proved to be an effective and efficient recruitment approach. Eighty-nine percent of those approached participated in the interviews in regional locations. This participation rate is higher than those in other street intercept studies [33, 34], possibly because the topic matter was relevant to most individuals and not perceived to be sensitive. However, the study was based in one state, and therefore, the findings may not be representative for the broader population.

This study has overcome a series of limitations that characterized earlier public opinion surveys conducted in Australia. In these studies, sociodemographic characteristics and political affiliation were often not reported, and high nonresponse rates were evident, suggesting participant selection bias, with respondents more likely to be interested in nutrition policy than nonrespondents [26]. Previous qualitative studies have tended to rely on focus groups and citizen juries, formats known to be methodologically problematic due to selection and social desirability bias [59, 60]. Finally, many previous studies did not involve regional participants [26].

## Conclusions

This study demonstrated that the level of support for nutrition policies generally among the sampled population was moderate to high, although nutrition policies perceived to be most intrusive to personal freedoms were the least popular amongst the general public. We found two major competing narratives. According to the more frequent narrative, people consume unhealthy foods and drinks because of a lack of knowledge about healthy diets. This narrative correlates strongly with the ideas of individual responsibility and personal freedoms, and disregards the commercial determinants of health. The alternative, less common narrative notes that large food corporations shape food environments and consumption habits, and thus are responsible for the poor dietary choices of the population. Interestingly, children and farmers were most often identified as “victims” by participants in this research. Public health policy-makers may be able to increase public support for regulatory nutrition policies by adopting framing that emphasizes protecting children and benefiting farmers.

## Supplementary Information

**Additional file 1.** Interview Guide.

## Data Availability

The datasets used and/or analysed during the current study are available from the corresponding author on reasonable request.
