# Supplementary material for: Competing public narratives in nutrition policy: insights into the ideational barriers of public support for regulatory nutrition measures
Source: Health Res Policy Syst. 2022 Aug 9;20:86. doi: 10.1186/s12961-022-00891-6 (PMC9361541; doi:10.1186/s12961-022-00891-6)
Supplement: Supplementary file 1 — Additional file 1. Interview Guide. [file 12961_2022_891_MOESM1_ESM.docx]

**Supplementary File 1 _ Interview Guide**

1) Do you think governments should get involved in making it easier for the public to consume heathier food and drinks?

2) If yes, why do you think the government should get involved? What do you think the government should do?

3) If no, why not?

4) (Regardless of previous answer) I’m going to read a list of possible things the government could consider implementing, could you tell us which ones you think would make the greatest impact to the health of Australians?

| **Policy Options** | Strongly Strongly  disagree agree | | | | |
| --- | --- | --- | --- | --- | --- |
| A. Ban vending machines selling unhealthy food or drinks in schools | 1 | 2 | 3 | 4 | 5 |
| B. Impose a tax on manufacturers for the high-sugar drinks they sell | 1 | 2 | 3 | 4 | 5 |
| C. Ban advertising of junk food targeting children during popular TV viewing times (including 6-9pm) | 1 | 2 | 3 | 4 | 5 |
| D. Subsidise the sales of fruits and vegetables, making them cheaper for consumers | 1 | 2 | 3 | 4 | 5 |
| E. Conduct media campaigns to encourage people to eat healthier foods, like fruit and vegetables | 1 | 2 | 3 | 4 | 5 |
| F. Encourage food companies to provide food labels that carry clearer information about the nutrition content of foods | 1 | 2 | 3 | 4 | 5 |
| G. Make companies reformulate foods to contain less salt, sugar and saturated fat | 1 | 2 | 3 | 4 | 5 |
| H. Provide freight subsidies from the government for healthy food to remote Aboriginal communities | 1 | 2 | 3 | 4 | 5 |
| I. Introduce a 20% tax on sugary drinks that would increase the price for consumers | 1 | 2 | 3 | 4 | 5 |

5) Is there anything missing here or do you have any other comments you would like to add?

6) We noticed that you are highly supportive of xx, could you tell us why?

7) We noticed that you are not supportive of xxx, could you tell us why?
